# Using social media to facilitate medical students’ interest in research

**DOI:** 10.3402/meo.v19.25860

**Published:** 2014-10-16

**Authors:** Abdulrahman A. Al-Khateeb, Hanan Y. Abdurabu

**Affiliations:** College of Medicine, Alfaisal University, Riyadh, Kingdom of Saudi Arabia

Of late, the use of social media has spread worldwide and there is a massive number of international users. According to reports from Facebook (1) and Twitter (2) during the second quarter of 2014, the number of active users reached 1.32 billion and 271 million, respectively. That being said, several studies have reported overwhelming responses from medical students using social media networking websites – especially Facebook (3–5).

Existing studies indicate a positive correlation between integration of social media into medical education and medical students’ attitudes toward acquiring knowledge and improving their skills (6). In addition, one study sought to evaluate the effectiveness of an online teaching forum – suggesting that it may enhance peer-to-peer teaching and answering each other's queries (7). From a clinical perspective, social media usage can play a central role in promoting awareness of early screening for diseases and in informing patients about the latest treatment or alternatives (8).

Medical students’ participation in undergraduate research (UR) is an exceedingly important part of the professionalization process, as students become adept at using the literature, generating innovative ideas, and enhancing their abilities to communicate (9). From our experience at the College of Medicine in Alfaisal University, where students have created a Facebook group to share their experiences and concerns, we believe social media boost medical students’ interest to participate in UR and generate transformative ideas.

In addition, there is another Facebook group that ‘gathers’ all medical students in a single, multi-operating platform – where junior and senior undergraduates can exchange valuable feedback, advice, and suggestions.

To the best of our knowledge, no formal studies have focused on the integration of social media and UR. For this reason, we would like to suggest several ways in which social media might be used to cultivate medical students’ research interests. First, creating a group on a social media website facilitates communication among members of the research project; it also provides a platform whereby mentors can assign tasks, address concerns, and inform project members of the latest developments. Second, another virtual group could be created to guide undergraduate students seeking to create new ideas and start their own projects. This provides a platform for discussion, answering concerns and enabling students to tackle obstacles they face as beginners.

Third, medical students can also use the Facebook group created by their corresponding batch to post links to their online-surveys and collect project data and elicit responses from their peers. This may encourage survey-based research to assess medical students’ attitudes and perspectives on education-related topics, for example. Finally, a private UR group can be created to include all medical students – where they can share with colleagues their accomplishments, achievements, and outcomes of their research experiences. Ultimately, this exchange may further promote interest in research among trainees of all levels.

## Conflicts of interest and funding

The authors have not received any funding or benefits from industry or elsewhere to conduct this study.

*Abdulrahman A. Al-Khateeb* College of Medicine Alfaisal University Riyadh Kingdom of Saudi Arabia Email: aal-khateeb@alfaisal.edu *Hanan Y. Abdurabu* College of Medicine Alfaisal University Riyadh Kingdom of Saudi Arabia

